# Right-ventricular assessment using a segmented cine acquisition employing iterative SENSE reconstruction with spatio-temporal L1 regularization: Initial clinical experience

**DOI:** 10.1186/1532-429X-18-S1-W18

**Published:** 2016-01-27

**Authors:** Abraham Bogachkov, Jad Bou Ayache, Bradley D Allen, Ian G Murphy, Maria L Carr, Michaela Schmidt, Michael O Zenge, James C Carr, Jeremy D Collins

**Affiliations:** 1 grid.465264.7Feinberg School of Medicine, Northwestern University, Chicago, IL USA; 2 grid.465264.7Radiology, Northwestern University, Chicago, IL USA; 3 Siemens Healthcare, GmbH, Erlangen, Germany

## Background

Cardiac MR (CMR) has emerged as the gold standard for assessing right-ventricular (RV) size and function with cine acquisitions. However, dyspnea often accompanies RV dysfunction, limiting patient ability to breath-hold and consequently image quality at CMR. The application of a novel iterative reconstruction technique to segmented CMR cine acquisitions may enable higher acceleration factors, shortening image acquisitions, while maintaining image quality for RV assessment. The purpose of this study is to evaluate the clinical utility of a prototype iterative reconstruction algorithm utilizing spatio-temporal L_1_-regularization (iteratively reconstructed Sparse-SENSE) in the quantitative assessment of RV systolic function.

## Methods

9 healthy volunteers (44.3 ± 13.5 yrs) and 29 patients (54.3 ± 13.8 yrs) were scanned on a 1.5T scanner. Cine images were acquired in the 4-chamber and short-axis orientations using conventional generalized auto-calibrating partially parallel acquisition (GRAPPA) factor 2 acceleration ("GRAPPA 2"), a spatio-temporal undersampled TSENSE acquisition with factor 4 acceleration ("TSENSE 4"), and iteratively reconstructed Sparse SENSE with an acceleration factor of 4 ("IS-SENSE 4"). Quantitative assessment of RV function was performed by a single reviewer. A subset of 14 subjects was re-analyzed by the initial reviewer and a second reviewer to assess intraobserver and interobserver agreement. Two independent reviewers scored images for image quality, noise, and artifacts on a 5-point Likert scale. All continuous variables were analyzed using linear regression with associated R^2^ values. Comparison of acquisition techniques was performed using univariate analysis of variance (ANOVA), and an intraclass correlation coefficient (ICC) was calculated to assess both intraobserver and interobserver agreement.

## Results

**Figure 1:**
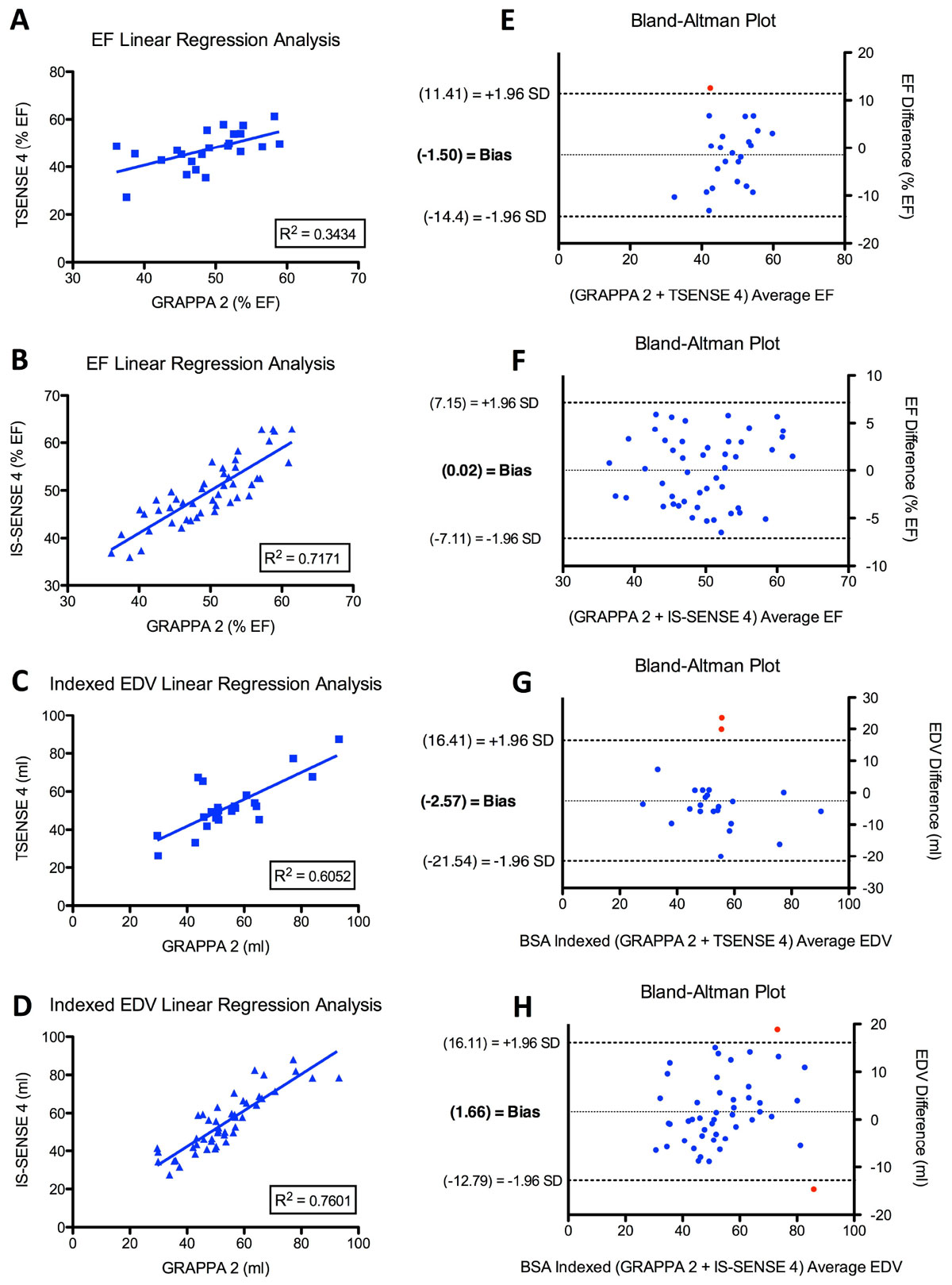
**A: linear regression analysis of TSENSE 4 versus GRAPPA 2 for RV EF** **B:** linear regression analysis of IS-SENSE 4 versus GRAPPA 2 for RV EF **C:** linear regression analysis of TSENSE 4 versus GRAPPA 2 for BSA indexed RV EDV **D:** linear regression analysis of IS-SENSE 4 versus GRAPPA 2 for BSA indexed RV EDV **E:** Bland-Altman plot comparing TSENSE 4 and GRAPPA 2 RV EF **F:** Bland-Altman plot comparing IS-SENSE 4 and GRAPPA 2 RV EF **G:** Bland-Altman plot comparing TSENSE 4 and GRAPPA 2 BSA indexed RV EDV **H:** Bland-Altman plot comparing IS-SENSE 4 and GRAPPA 2 BSA indexed RV EDV. Red points indicate averages outside of 1.96 SD. **EF** = ejection fraction; **EDV** = end diastolic volume

**Figure 2:**
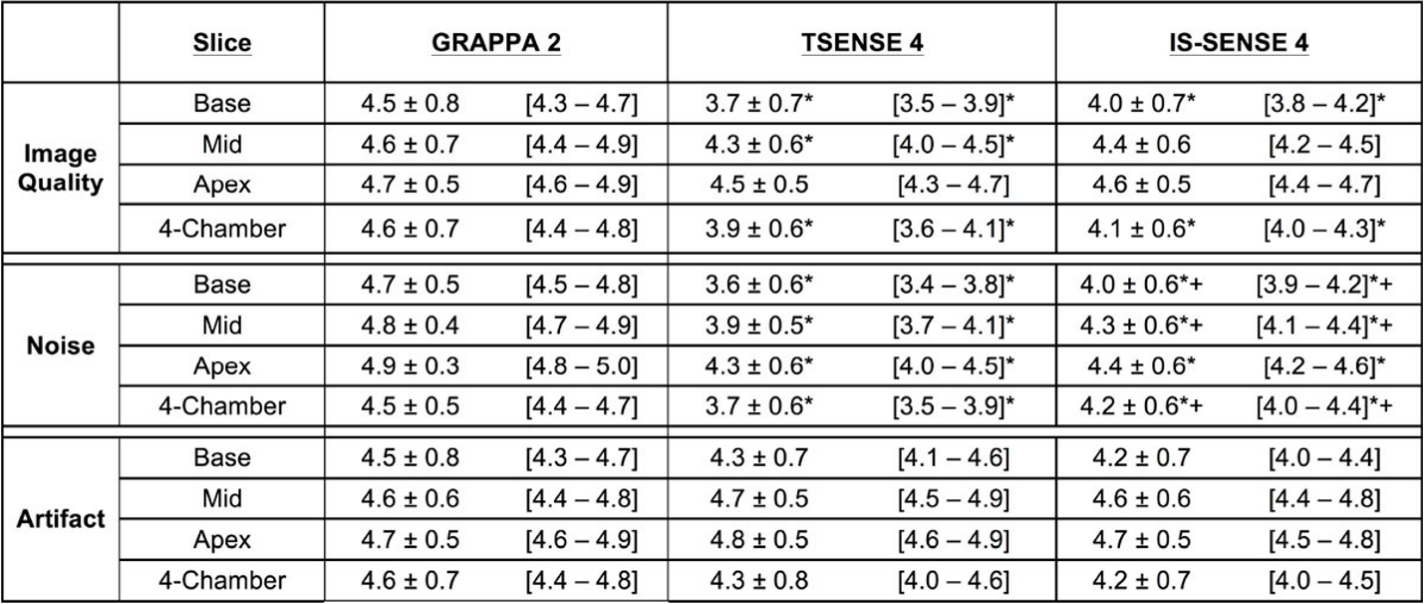
**Slice-by-Slice qualitative comparison between each acceleration approach**. Data are presented as mean ± standard deviation [corrected 95% confidence interval]. * = statistically significant (p < 0.05) difference from GRAPPA 2. + = statistically significant (p < 0.05) difference from TSENSE 4.

Among all three acquisition types, differences in RV ejection fraction (EF) and BSA-indexed end-diastolic volume (EDVi) were shown to be statistically insignificant. The R^2^ values for linear regression of TSENSE 4 and IS-SENSE 4 versus GRAPPA 2 were 0.34 and 0.72 for RV EF, and 0.61 and 0.76 for RV EDVi, respectively (Figure 1). ICC results for both intraobserver and interobserver consistency between GRAPPA 2 and IS-SENSE 4 yielded excellent agreement. Qualitative review yielded small, but statistically significant differences (p < 0.05) in image quality and noise between both TSENSE 4 and IS-SENSE 4 compared to GRAPPA 2 (Figure 2). All three techniques were rated as nearly artifact free.

## Conclusions

2D CINE imaging acquisitions with prototype IR Sparse-SENSE reconstruction and an acceleration factor of 4 accurately and reliably quantitates RV systolic function parameters, while maintaining image quality comparable to segmented cine sequences with GRAPPA factor 2 acceleration. IR Sparse-SENSE has the potential to improve imaging options in patients with intermittent arrhythmias or difficulties with breath holding.

